# New Insights into the Pathogenesis of Experimental Cytomegalovirus Retinal Necrosis with an Emphasis on Inflammasomes and Pyroptosis

**DOI:** 10.3390/pathogens14090879

**Published:** 2025-09-03

**Authors:** Richard D. Dix, Jessica J. Carter, Heather Koehler, Hongyan Guo

**Affiliations:** 1 Viral Immunology Center, Department of Biology, Georgia State University, Atlanta, GA 30303, USA; jcarter80@gsu.edu; 2 Department of Ophthalmology, Emory University School of Medicine, Atlanta, GA 30322, USA; 3 School of Molecular Biosciences, College of Veterinary Medicine, Washington State University, Pullman, WA 99164, USA; heather.koehler@wsu.edu; 4 Department of Microbiology and Immunology, Louisiana State University, Shreveport, LA 71103, USA; hongyan.guo@lsuhs.edu

**Keywords:** inflammasomes, pyroptosis, necroptosis, murine cytomegalovirus, MAIDS, retinitis, human cytomegalovirus, AIDS

## Abstract

Pyroptosis is a programmed cell death pathway that initiates and sustains inflammation to protect the host against invading pathogens or stress. Activation of caspase-1-mediated canonical pyroptosis takes place via formation of multi-protein cytoplasmic immune signaling complexes known as inflammasomes. Because we have shown previously that the canonical pyroptosis pathway plays a significant role in the pathogenesis of experimental murine cytomegalovirus (MCMV) retinal necrosis in mice with retrovirus-induced immunosuppression (MAIDS), we performed additional studies to determine whether this pathogenic involvement extends to inflammasomes as initiators of the canonical pyroptosis pathway. Initial studies demonstrated significant transcription of three different pyroptosis-associated inflammasomes, NLRP3, NLRP1b, and AIM2, within the ocular compartments of MCMV-infected eyes of MAIDS mice. Subsequent histopathologic findings revealed MCMV-infected eyes of groups of NLRP3^−/−^ MAIDS mice, NLRP1b^−/−^ MAIDS mice, or AIM2^−/−^ MAIDS mice each exhibited a similar atypical retinal pathology characterized by loss of photoreceptors and proliferation and/or loss of retinal pigmented epithelium but with relative sparing of the neurosensory retina, an outcome different from typical full-thickness retinal necrosis of MCMV-infected eyes of wildtype MAIDS mice. We conclude that multiple inflammasomes are individually stimulated within MCMV-infected eyes of MAIDS mice and each independently contributes to MAIDS-related MCMV full-thickness retinal necrosis pathogenesis.

## 1. Introduction

The retina of the eye is susceptible to significant pathologies of viral origin, with a majority caused by human herpesviruses. Of these, human cytomegalovirus (HCMV) has appeared as the primary etiologic agent for a retinal necrosis in immunosuppressed patients, including AIDS patients [1]. At the outset of the AIDS pandemic, approximately 30% of this patient population developed HCMV retinal necrosis [2]. Its incidence has fallen sharply, however, due to the development of antiretroviral therapy (ART) to manage HIV-induced immunosuppression. Persons without access to ART or those who fail to respond to ART nonetheless continue to develop this sight-threatening disease. Thus, AIDS-related HCMV retinal necrosis remains a serious ophthalmologic problem worldwide and has been called “the neglected disease of the ongoing AIDS pandemic” [3].

Although cellular immunity is significantly diminished during the evolution of AIDS-related HCMV retinal necrosis, several components of innate immunity continue to operate. This is suggested by an influx of neutrophils and macrophages within foci of retinal tissue undergoing necrosis [4,5]. This observation suggests that other components of innate immunity may also operate. These may include regulated or programmed cell death (PCD) pathways such as apoptosis, pyroptosis, and necroptosis (Table 1), which serve as important innate immunity mechanisms to protect the host against invading pathogens or stress. Of these, pyroptosis is a caspase-1-mediated PCD that is uniquely associated with the stimulation of a pronounced and sustained inflammation [6], although other caspases, such as caspase-11 in mice and caspase-4 and caspase-5 in humans [7,8], have been shown to trigger pyroptosis through a noncanonical pathway [9].

During canonical pyroptosis, pro-caspase-1 is cleaved to yield caspase-1, which, in turn, cleaves gasdermin D (GSDMD). GSDMD is a cytoplasmic protein whose subunit localizes to the plasma membrane, where it forms multiple pores [12,13]. These pores result in a net increase in osmotic pressure, water influx, cell swelling, and eventually osmotic lysis, which leads to the release of two proinflammatory cytokines, interleukin-1β (IL-1β) and interleukin-18 (IL-18) [14]. These proinflammatory cytokines ultimately cause the hallmark inflammation associated with pyroptosis and thereby serve as critical mediators of host innate immune responses against invading pathogens.

Activation of caspase-1-mediated pyroptosis takes place via multi-protein cytoplasmic immune signaling complexes known as inflammasomes [15]. Formation of functional inflammasomes is initiated through pattern recognition receptors (PRRs), which serve as pathogen or stress sensors that recognize pathogen-associated molecular patterns (PAMPs) and danger-associated molecular patterns (DAMPs), respectively. Upon sensing a specific PAMP or DAMP, PRRs recruit an adapter protein apoptosis-associated speck-like protein (ASC), which contains a caspase activation and recruitment domain (CARD) in addition to caspase-1. Thus, a functional inflammasome complex consists of the activated inflammasome sensor, ASC, and caspase-1 [15]. It is within this complex that caspase-1 catalyzes the proteolytic cleavage of GSDMD that ultimately leads to the release of proinflammatory cytokines IL-1b and IL-18.

The assembly of distinct inflammasome complexes is dependent on which PRR is activated. During caspase-1-mediated pyroptosis, these typically are members of the leucine-rich repeat (LRR)-containing protein (NLR) family, which includes NLRP1 and NLRP3 [16]. NLRP1 was the first member of the NLR protein family recognized to form an inflammasome complex [17]. NLRP1b, a murine paralogue of NLRP1 [18], has been shown to be activated in response to a lethal toxin produced by the bacterium *Bacillus anthracis*, but other stimuli for NLRP1b activation are possible [15]. In comparison, NLRP3 is activated in response to diverse stimuli, including bacteria, viruses, and fungi [19,20]. In sharp contrast to inflammasomes of the NLR protein family, absent in melanoma 2 (AIM2) is not NLR-based and senses and binds to foreign cytoplasmic double-stranded DNA of cytosolic bacteria and DNA viruses [21], including cytomegaloviruses [22].

To explore a possible role for pyroptosis during the pathogenesis of AIDS-related HCMV retinal necrosis, we have been using a well-characterized mouse model of experimental murine cytomegalovirus (MCMV) retinal necrosis that develops in mice with murine acquired immunodeficiency syndrome (MAIDS) by 10 days after intraocular (subretinal) MCMV inoculation [23,24]. MCMV has often been the cytomegalovirus of choice to investigate various aspects of the pathogenicity of HCMV due to striking similarities in their genomic structures, immunology, latency, and cellular/tissue tropisms [25]. Other mouse models of experimental cytomegalovirus retinal necrosis using MCMV have included mice immunosuppressed with corticosteroids [26,27], but drug immunosuppression does not reproduce the unique immunopathologic evolution of AIDS following HIV infection. In comparison, MAIDS is a progressive immunodeficiency of C57BL/6 mice induced by a mixture of mouse retroviruses that has been shown by others previously [28,29,30] to be a clinically relevant animal model to study AIDS pathogenesis. In fact, MAIDS shares many immunopathologic features with AIDS. These immunopathologic features include the appearance of chronic generalized lymphadenopathy, polyclonal B-cell activation, hypergammaglobulinemia, a Th1 to Th2 shift in cytokine production, and ultimately severe deficiencies in functions of both CD4+ and CD8+ T-cells functions [28,29,30]. Moreover, 80 to 100% of MCMV-infected eyes of mice with MAIDS exhibit histopathologic features that mimic those found in the eyes of patients with AIDS-related HCMV retinal necrosis. These histopathologic features include the development of prominent cytomegalic cells contained with areas of full-thickness retinal necrosis, i.e., all areas of the retina show complete destruction of the retinal architecture [23,24].

Using the MAIDS model of MCMV retinal necrosis, we have shown previously that MCMV-infected eyes of mice with MAIDS produce significantly high amounts of caspase-1, GSDMD, IL-1β, and IL-18 transcripts, as well as translated protein products within the ocular compartment during the onset and development of retinal disease [31,32]. Studies using mice with MAIDS deficient in either caspase-1, GSDMD, or IL-18, and therefore deficient in the canonical pyroptosis pathway, have provided additional evidence that this PCD contributes to the pathogenesis of full-thickness retinal necrosis, which consistently develops in this animal model of AIDS-related HCMV retinal necrosis [32].

The present investigation serves as a review, as well as a report on our ongoing studies to define with greater clarity the precise role of pyroptosis during MAIDS-related MCMV retinal necrosis pathogenesis, with a focus on pyroptosis-associated inflammasomes. Herein, we show that MAIDS mice deficient in either the NLRP3, NLRP1b, or AIM2 inflammasome share a pattern of atypical MCMV retinal disease that is similar, if not identical, to that observed in MCMV-infected MAIDS mice deficient in key components of the canonical pyroptosis pathway, i.e., caspase-1, GSDMD, or IL-18 [32]. We conclude that the NLRP3, NLRP1b, and AIM2 inflammasomes of canonical pyroptosis individually contribute to the full-thickness retinal necrosis typically observed during the pathogenesis of MAIDS-related MCMV retinal necrosis.

## 2. Materials and Methods

### 2.1. Animals

Adult female wildtype (WT) C57BL/6 mice, adult female mice deficient in NLRP3 (B6.129S6-*nlrp3*^tm3Hhf^/J), adult female mice deficient in NLRP1b (B6.129S6-*Nlrp1b*^tm1Bhk^/J), and adult female mice deficient in AIM2 (B6.129P2-Aim2^Gt(CSG445)Byg^/J) were purchased from Jackson Laboratory (Bar Harbor, ME, USA). Adult female WT BALB/c mice were purchased from Harlan Laboratories (Indianapolis, IN, USA). Female mice were used throughout the investigation because sex-linked differences have not been reported for AIDS-related HCMV retinitis in humans [1,2]. All mice were housed in the AAALAC-accredited Georgia State University (GSU) vivarium and maintained on alternative 12 hr light–dark cycles with unrestricted access to standard diet and water. All animal procedures were approved by the Georgia State University Institutional Animal Care and Use Committee (IACUC) (Protocol A25033).

### 2.2. Viruses

Virus stocks of the Smith strain of MCMV were prepared in salivary glands of BALB/c mice as described previously [32]. This was accomplished via intraperitoneal injection with approximately 10^2^ to 10^3^ plaque forming units (PFUs) of MCMV contained within a 0.2 mL volume of Dulbecco’s modified Eagle’s medium (DMEM) (Corning, Corning, NY, USA) containing 10% fetal bovine serum. Salivary glands were harvested aseptically 14 days later, pooled, homogenized in DMEM, clarified by centrifugation, and stored in liquid N_2_ in 0.5 mL aliquots. Each experiment was performed using a fresh aliquot of MCMV stock.

Stocks of murine retrovirus (LP-BM5 MuLV) [32] were prepared in cultures of SC-1 fibroblasts (ATCC, Manassas, VA, USA) and SC-1/MuLV LP-BM5 cells (kindly provided by the AIDS Research and Reference Reagent Program, Division of AIDS, NIAID, NIH, Germantown, MD, USA) at a 1:1 ratio and maintained in DMEM. Six days later, the cells were scraped into the DMEM, pelleted by centrifugation, resuspended in phosphate-buffered saline (PBS), and aliquots were stored at −80 °C. Each aliquot was thawed and clarified by centrifugation prior to use.

### 2.3. Induction of MAIDS

Induction of MAIDS was accomplished via intraperitoneal injection of 1.0 mL of LP-BM5 MuLV (~5 × 10^3^ to 5 × 10^4^ of infectious murine retrovirus) in groups of WT C57BL/6 mice and groups of mice deficient in NLRP3, NLRP1b, or AIM2 [32]. Mice with MAIDS of 4 weeks’ duration (MAIDS-4 mice) and 10 weeks’ duration (MAIDS-10 mice) [33] were used in studies described in Section 3.1 of the Results, whereas Section 2.5, Section 2.6, Section 2.7, Section 2.8, Section 2.9, Section 3.1 and Section 3.2 of the Results used only MAIDS of 10 weeks’ duration (MAIDS-10 mice).

### 2.4. Intraocular Injection of MCMV

Following administration of anesthesia, the left eyes of all groups of mice with MAIDS were inoculated by intraocular (subretinal) injection with approximately 10^4^ PFU of MCMV [Smith] contained within a 2 uL volume of DMEM to induce retinal disease. The contralateral right eyes were injected intraocularly (subretinally) with DMEM alone and served as internal controls for each animal, because these eyes do not develop retinal disease. Details of the subretinal inoculation procedure have been summarized previously [32].

### 2.5. Quantification of Infectious MCMV

Whole MCMV-infected eyes from all groups of mice with MAIDS were collected at 10 days after intraocular virus injection because most severe MCMV-induced retinal necrosis is observed at this time postinfection [10]. All eyes were stored at −70 °C prior to quantification of amounts of intraocular infectious virus for each eye by standard quantitative plaque assay. Whole eyes were thawed, individually homogenized in 1.0 mL of cold DMEM, and clarified by centrifugation. Individual eye homogenates were then subjected to tenfold dilutions in cold DMEM and titered in duplicate onto monolayers of mouse embryo fibroblasts (ATCC, Manassas, VA, USA) contained within six-well plates. Following a 1 h adsorption at 37 °C, the virus-inoculated cell monolayers were overlaid with 2% methylcellulose containing DMEM, incubated for 6 days at 37 °C in a humidified CO_2_ atmosphere, and quantified for individual plaque formation using an inverted light microscope. Results were reported as PFU/mL/eye.

### 2.6. Quantitative Real-Time Reverse Transcriptase PCR (RT-PCR) Assay

Whole MCMV-infected eyes and whole contralateral mock-infected eyes (control) were collected from WT mice at 3, 6, and 10 days postinoculation, which represent key time points during the onset and progression of MAIDS-related MCMV retinal necrosis [23]. All eyes were stored at −70 °C in RNAlater solution (Ambion, Austin, TX, USA). For analysis, whole eyes were thawed and individually homogenized in 1.0 mL of TRIzol^®^ reagent (Invitrogen Life Technologies, Carlsbad, CA, USA), and total RNA was extracted from each eye homogenate using chloroform and purified using the PureLink^®^ RNA Mini Kit according to the manufacturer’s instructions (Ambion/ThermoFisher, Waltham, MA, USA). Following determination of total RNA amounts using a SmartSpec 3000 spectrometer (Bio-Rad Laboratories, Hercules, CA, USA), the total RNA concentration was normalized for each sample, and cDNA synthesis was performed using the SuperScript^™^ III First-Strand Synthesis Kit reagents according to the manufacturer’s instructions (Invitrogen/ThermoFisher, Waltham, MA, USA). Specific primers for each mouse inflammasome under investigation were used to detect and quantify gene expression for each gene of interest. This was accomplished using each specific primer and the Power SYBR Green Master mix (Applied Biosystems, Foster City, CA, USA) in combination with Applied Biosystems 7500 Fast Real-Time PCR System instrumentation with detection system software 2.0 (Applied Biosystems, Foster City, CA, USA). Mouse-specific primers for NLRP3 (QT00122458), NLRP1b (QT01772183), AIM2 (QT01755404), and glyceraldehyde-3-phosphate dehydrogenase (GAPDH) (QT01192646) were purchased from Qiagen (Valencia, CA, USA). Samples were analyzed for 10 min at 95 °C, followed by 40 cycles consisting of 15 s at 94 °C, 31 s at 55 °C, and 35 s at 70 °C. Cycles to threshold (C_T_) for each target gene were determined, the ΔC_T_ value for each sample was normalized by subtracting the C_T_ value of their own endogenous housekeeping gene (GAPDH) from the C_T_ value of the target gene, and the ΔC_T_ values of each target gene mRNA of MCMV-infected eyes were compared with mock-infected control eyes by the 2^−ΔΔCt^ method to determine the change in gene expression, thereby yielding a relative fold change in mRNA expression for each group.

### 2.7. Western Blot Assay

Whole MCMV-infected eyes and whole contralateral mock-infected eyes (control) were collected from groups of MAIDS-10 mice deficient in either NLRP3, NLRP1b, or AIM2 genes or WT mice at 10 days postinoculation, stored in liquid N_2,_ thawed, and homogenized in PBS containing a protease inhibitor cocktail (Sigma, St. Louis, MO, USA). Standard Western blot analysis was performed for detection of RIPK1 and RIPK3 proteins using rabbit anti-mouse RIPK1 antibody (1:500) or rabbit anti-mouse RIPK3 antibody (1:500) (Abcam, Cambridge, UK) as primary antibodies. Goat anti-rabbit IgG antibody (heavy plus light chains) conjugated with horseradish peroxidase (Thermo Scientific, Pittsburgh, PA, USA) was chosen as the secondary antibody (1:5000). Probed nitrocellulose membranes (Bio-Rad, Hercules, CA, USA) were treated with Clarity Western ECL Substrate (Bio-Rad, Hercules, CA, USA) and exposed to HyBlot film (Denville, Holliston, MA, USA) for band detection.

### 2.8. Histopathologic Analysis

All histopathologic preparation of whole MCMV-infected eyes was performed at the Department of Pathology, Emory Eye Center, Emory University School of Medicine. Following careful removal from euthanized animals, all eyes were fixed in 10% buffered formalin for at least 5 days at 4 °C, embedded in paraffin, cryostat-cut into 5 μm thick transverse sections, and stained with hematoxylin and eosin. As previously reported [32], every sixth section of each eye was scored by light microscopy for the presence or absence (frequency) of full-thickness retinal necrosis or evaluated for possible atypical retinal pathology. Eye sections were viewed and photographed using a Nikon Ellipse 50i microscope with camera attachment.

### 2.9. Statistical Analysis

Statistical analyses were performed using GraphPad Prism v6.07 software with a significance level (α) set to 0.05; *p*-values of <0.05 were considered statistically significant. At least two independent experiments were performed for each study, and the mean and standard deviation for each group were calculated. For quantitative real-time RT-PCR assays, statistical analysis was performed by comparing MCMV-infected eyes with mock-infected eyes (controls) by paired, two-tailed Student’s *t*-test. For comparing ocular titers, statistical analysis was performed by comparing MCMV-infected eyes of WT MAIDS-10 mice to MCMV-infected eyes of MAIDS-10 mice deficient in either NLRP3, NLRP1b, or AIM2 genes by unpaired two-tailed Student’s *t*-test.

## 3. Results

### 3.1. MCMV-Infected Eyes of Mice with MAIDS-10 Show Significant Stimulation of NLRP3, NLRP1b, and AIM2 Transcripts When Compared with MCMV-Infected Eyes of MAIDS-4 Mice

**Figure 1:**
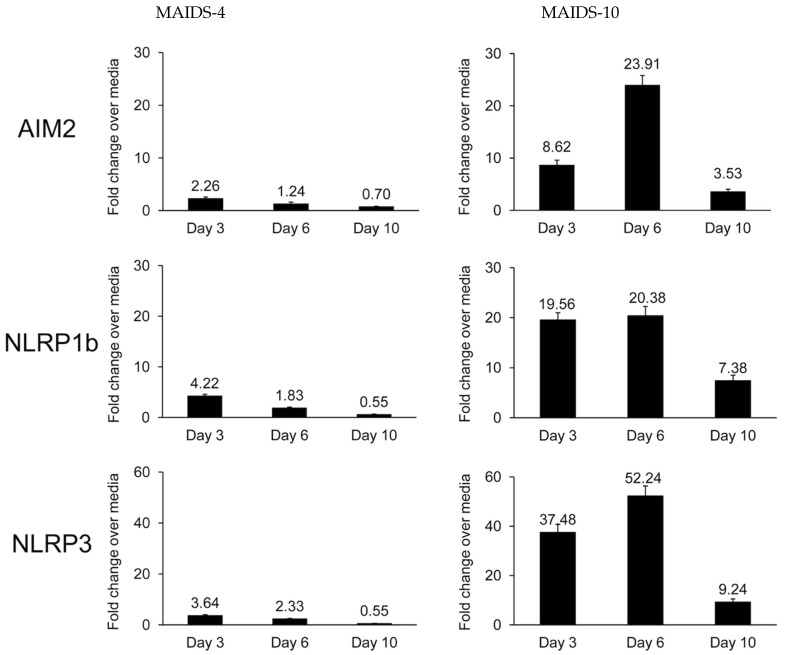
Detection of AIM2, NLRP1b, and NLRP3 transcripts in MCMV-infected eyes collected from groups of MAIDS-4 mice (*n* = 5) and MAIDS-10 mice (*n* = 5) at 3, 6, and 10 days after subretinal MCMV injection when compared with contralateral mock-infected eyes (controls). Levels (fold-change) were determined by quantitative RT-PCR assay. Bars = standard error.

During the course of previous investigations, we discovered that MCMV-infected eyes of mice with MAIDS of 4 weeks’ duration (MAIDS-4 mice) are resistant to the development of full-thickness retinal necrosis although pronounced proliferation of RPE is often noted [33]. In sharp contrast, 80–100% of MCMV-infected eyes of mice with MAIDS of 10 weeks’ duration (MAIDS-10 mice) are susceptible to the development of full-thickness retinal necrosis [32,33]. We therefore initially compared MCMV-infected eyes of groups of retinitis-resistant MAIDS-4 mice with the MCMV-infected eyes of groups of retinitis-susceptible MAIDS-10 mice for detection and quantification of NLRP3, NLRP1b, or AIM2 transcripts. The results are shown in Figure 1. MCMV-infected eyes from retinitis-susceptible MAIDS-10 mice showed significant amounts of mRNAs by quantitative RT-PCR assay for all three inflammasomes when compared with the contralateral mock-infected eyes (controls) at 3, 6, and 10 days after intraocular MCMV infection. High amounts of intraocular mRNA production were consistently noted for the NLRP3, NLRP1b, and AIM2 inflammasomes at 3 and 6 days postinfection. Peak amounts were observed at 6 days postinfection, but with amounts dramatically decreasing by 10 days postinfection. As predicted, intraocular amounts of mRNAs for all three inflammasomes within MCMV-infected eyes of retinitis-resistant MAIDS-4 mice were comparatively low at all days examined although minor stimulation of transcripts was observed consistently at 3 days postinfection. We have noted similar minor levels of transcription at 3 days postinfection in previous studies investigating the contributions of parthanatos [34] and IL-1α [35] toward MAIDS-related MCMV retinal necrosis. We postulate without experimental evidence that this occurrence is due to a nonspecific low level of intraocular inflammation that takes place in response to intraocular trauma induced by needlestick alone.

### 3.2. MCMV-Infected Eyes of MAIDS-10 Mice Deficient in NLRP3, NLRPb, or AIM2 Fail to Develop Full-Thickness Retinal Necrosis as Occurs Within MCMV-Infected Eyes of MAIDS-10 Wildtype Mice

We next investigated a possible mechanistic role for each of these inflammasomes during the pathogenesis of MAIDS-related MCMV retinal necrosis. This was accomplished using mice with MAIDS-10 deficient in the production of either NLRP3, NLRP1b, or AIM2. Accordingly, MAIDS-10 was induced in groups of NLRP3^−/−^ mice, NLRP1b^−/−^ mice, AIM2^−/−^ mice, and wildtype mice (control) via intraperitoneal inoculation with the immunosuppressive murine retrovirus mixture LP-BM5 [32]. Importantly, loss of function of an individual inflammasome gene did not prevent development of MAIDS in either NLRP3^−/−^ mice, NLRP1b^−/−^ mice, or AIM2^−/−^ mice, as determined by the appearance in all animals of a prominent generalized lymphadenopathy characteristic of MAIDS by 10 weeks after retrovirus inoculation, as reported by us previously [32].

The eyes of groups of NLRP3^−/−^ MAIDS-10 mice, NLRP1b^−/−^ MAID-10 mice, AIM2^−/−^ MAIDS-10 mice, and wildtype MAIDS-10 mice in separate experiments were infected intraocularly with MCMV, collected at 10 days postinfection, and scored for frequency of full-thickness retinal necrosis following histopathologic analysis. As expected [32,33], MCMV-infected eyes of 89% of wildtype mice with MAIDS-10 developed full-thickness retinal necrosis (Table 2). In sharp contrast, none (0%) of MCMV-infected eyes of MAIDS-10 deficient in either the NLRP3, NLRP1b, or AIM2 inflammasome exhibited typical full-thickness retinal necrosis as seen in MCMV-infected wildtype MAIDS-10 mice.

Whole MCMV-infected eyes were collected from groups of wildtype mice with MAIDS-10 and groups of mice with MAIDS-10 deficient in either NLRP3 (NLRP3^−/−^ MAIDS-10), NLRP1b (NLRP1b^−/−^ MAIDS-10), or AIM2 (AIM2^−/−^ MAIDS-10) at 10 days after subretinal MCMV inoculation, subjected to histopathologic analysis, and scored for full-thickness retinal necrosis (retinal necrosis/total).

### 3.3. MCMV-Infected Eyes of MAIDS-10 Mice Deficient in NLRP3, NLRPb, or AIM2 All Show an Atypical Pattern of Retinal Disease

**Figure 2:**
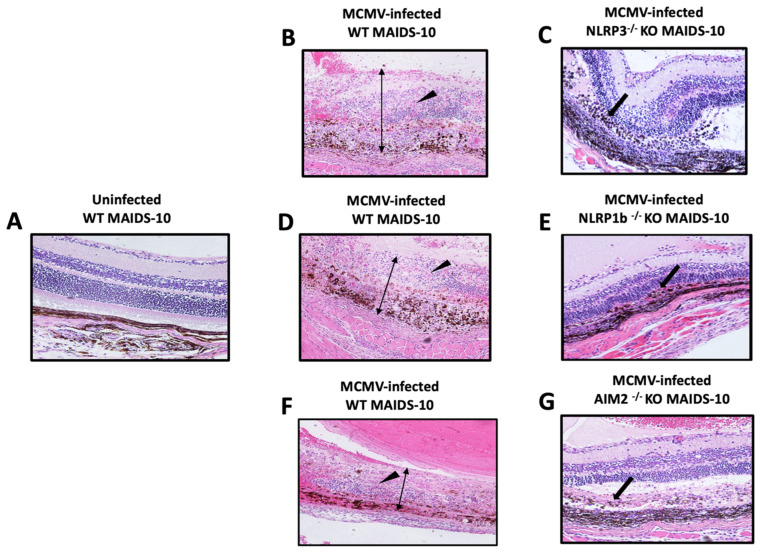
MCMV-infected eyes from groups of MAIDS-10 mice deficient in pyroptosis-associated inflammasomes show an atypical retinal pathology when compared with the MCMV-infected eyes of wildtype (WT) MAIDS-10 mice (*n* = 4–9). MCMV-infected eyes were collected at 10 days postinfection from groups of WT MAIDS-10 mice (**B**,**D**,**F**), NLRP3^−/−^ KO (knockout) MAIDS-10 mice (**C**), NLRP1b^−/−^ KO MAIDS-10 mice (**E**), and AIM2^−/−^ KO MAIDS-10 mice (**G**) and subjected to histopathologic analysis following hematoxylin and eosin staining. Full-thickness retinal necrosis within sections of MCMV-infected eyes of WT MAIDS-10 mice is indicated by thin double arrow heads. Thickening and proliferation of the RPE layer within individual retinal sections of MCMV-infected eyes of NLRP3^−/−^ KO MAIDS-10 mice (**C**) and NLRP1b^−/−^ KO MAIDS-10 mice (**D**) or loss of the RPE layer some retinal sections of in AIM2^−/−^ KO MAIDS-10 mice (**G**) is indicated using thick black arrows. Cytomegalic cells within sections of MCMV-infected eyes of WT MAIDS-10 mice (**B**,**D**,**F**) are indicated using black triangular arrows. A section of retina from an uninfected and unmanipulated eye of a WT MAIDS-10 mouse showing normal retinal architecture is included for comparisons (**A**). Original magnification = 200×.

Because 100% of MCMV-infected eyes of all MAIDS-10 mice deficient in either NLRP3, NLRP1b, or AIM2 inflammasome production failed to show typical full-thickness retinal necrosis at 10 days postinfection, this prompted us to perform a more rigorous histopathologic analysis of all animal groups to define and compare their patterns of retinal disease in more detail. The results are shown in Figure 2. Histopathologic analysis of sections of retina collected from MCMV-infected wildtype MAIDS-10 mice showed multiple areas of full-thickness retinal necrosis as the expected outcome (Figure 2B,D,F). In comparison, histopathologic analysis of MCMV-infected eyes of groups of NLRP3^−/−^ MAIDS-10 mice, NLRP1b^−/−^ MAIDS-10, and AIM2^−/−^ MAIDS-10 mice all consistently showed a consistent and similar pattern of atypical retinal disease. Firstly, all MCMV-infected eyes of all MAIDS-10 mice deficient in either the NLRP3, NLRP1b, or AIM2 inflammasome showed loss of photoreceptor cells (Figure 2C,E,G). Secondly, retinal pigmented epithelial (RPE) cells showed either varying degrees of proliferation (Figure 2C,E) or were absent (Figure 2G) in individual retinal sections. Thirdly, and most importantly, MCMV-infected eyes of all MAIDS-10 mice deficient in either the NLRP3, NLRP1b, or AIM2 inflammasome all exhibited relative preservation of the neurosensory retina (Figure 2C,E,G), unlike MCMV-infected eyes of wildtype MAIDS-10 mice (Figure 2B,D,F).

Although this novel pattern of atypical retinal disease was observed among all groups of animals with MAIDS-10 deficient in each of the three inflammasome gene function, it could not be attributed to MAIDS of ten week’s duration (MAIDS-10). The unmanipulated eyes of these animal groups and the mock-infected eyes of these animal groups all consistently exhibited normal retinal architecture. It is also noteworthy that sections of MCMV-infected eyes of wildtype MAIDS-10 mice consistently exhibited prominent foci of cytomegalic cells (Figure 2B,D,F). This finding was not observed in sections of MCMV-infected MAIDS-10 mice deficient in either NLRP3, NLRP1b, or AIM2 inflammasome genes (Figure 2C,E,G).

### 3.4. MCMV-Infected Eyes of MAIDS-10 Mice Deficient in Either NLRP3, NLRP1b, or AIM Harbor Significant Amounts of Infectious Virus but Significantly Less than That Observed for MCMV-Infected Eyes of Wildtype MAIDS-10 Mice

**Figure 3:**
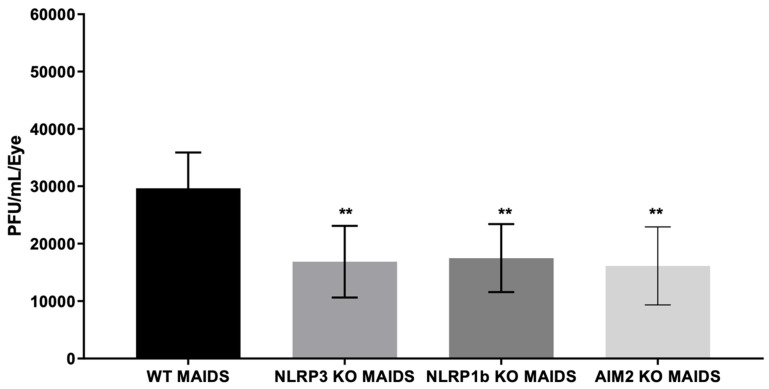
MCMV-infected eyes of NLRP3 KO (knockout) MAIDS-10 mice, NLRP1b KO MAIDS-10 mice, and AIM2 KO MAIDS-10 mice all harbor equivalent amounts of intraocular infectious MCMV but at ~50% of that found within MCMV-infected wildtype (WT) MAIDS-10 mice. Whole MCMV-infected eyes were collected at 10 days after intraocular MCMV inoculation from all groups of animals, individually homogenized, and individually subjected to standard plaque assay for quantification of infectious virus (*n* = 3–9). ** *p* <0.01.

We have shown that retinitis-susceptible MCMV-infected eyes of MAIDS-10 mice reproducibly harbor high amounts of infectious virus (~30,000 PFU/eye) at 10 days after intraocular inoculation [10,31] when compared with MCMV-inoculated eyes of healthy mice without MAIDS that fail to develop retinal disease (~250 PFU/eye) [10,31]. Due to the unusual pattern of retinal disease exhibited by all MCMV-infected eyes of MAIDS-10 mice deficient in each of the three inflammasomes under investigation, we were curious about the production of infectious virus within the MCMV-infected eyes of these animal groups. MCMV-infected eyes were therefore collected from groups of wildtype MAIDS-10 mice, NLRP3^−/−^ MAIDS-10 mice, NLRP1b^−/−^ MAIDS-10 mice, and AIM2^−/−^ MAIDS-10 mice at 10 days after intraocular MCMV inoculation and subjected to standard plaque assays for quantification and comparison of infectious MCMV within their globes. The results are shown in Figure 3. As predicted, MCMV-infected eyes collected from wildtype MAIDS-10 mice harbored ~30,000 PFU/eye. In comparison, MCMV-infected eyes collected from MAIDS-10 mice deficient in either the NLRP3, NLRP1b, or AIM2 inflammasome all harbored an equivalent amount of infectious virus but significantly reduced (~50%) when compared with MCMV-infected eyes of wildtype MAIDS-10 mice.

### 3.5. RIPK1 and RIPK3 of the Necroptosis Pathway Are Stimulated Within MCMV-Infected Eyes of MAIDS-10 Mice Deficient in Either NLRP3, NLRP1b, or AIM

**Figure 4:**
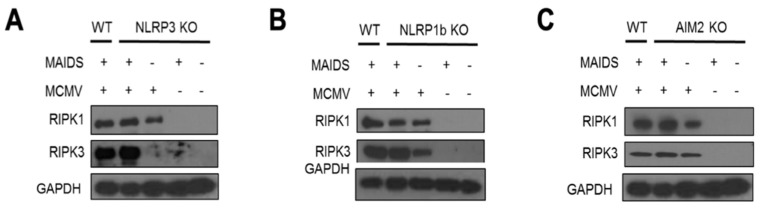
Necroptosis-associated RIPK1 and RIPK3 proteins are stimulated intraocularly at 10 days postinfection within the eyes of MCMV-infected eyes from groups of wildtype (WT) MAIDS-10 mice, (**A**) NLRP3 KO (knockout) MAIDS-10 mice, (**B**) NLRP1b KO MAIDS-10 mice, (**C**) AIM2 KO MAIDS-10 mice, and WT mice without MAIDS but deficient in each of the three inflammasomes. RIPK1 and RIPK3 proteins were not detected in groups of mock-infected eyes of mice with or without MAIDS-10 (*n* = 4–6). Whole eyes from each animal group were pooled and subjected to Western blot assay. The figure represents a composite of individual Western blot assays. GAPDH served as a control.

Necroptosis is another lytic and inflammatory PCD that, unlike pyroptosis, is caspase-independent [36] (Table 1). Instead, necroptosis is dependent upon recruitment of receptor-interacting protein kinase (RIPK)3, which may be initiated downstream by RIPK1 in response to death receptor signaling [37,38,39]. RIPK3, upon self-phosphorylation, stimulates oligomerization of mixed lineage kinase domain-like (MLKL) protein. MLKL protein then travels to the plasma membrane of the cell and forms pores, which ultimately leads to cell death through loss of membrane integrity and cell leakage of cellular contents, which become danger signals and induce inflammation [40,41]. Because we have already provided evidence for the operation of multiple PCD pathways during the pathogenesis of MAIDS-related MCMV retinal necrosis [10], we wondered whether necroptosis might continue to operate in the absence of pyroptosis-associated inflammasomes. MCMV-infected eyes and mock-infected eyes were collected from groups of wildtype MAIDS-10 mice, NLRP3^−/−^ MAIDS-10 mice, NLRP1b^−/−^ MAIDS-10 mice, and AIM2^−/−^ MAIDS-10 mice at 10 days after intraocular MCMV inoculation and subjected to Western blot analysis for detection of necroptosis-associated RIPK1 and RIPK3 proteins. Mock-infected eyes collected from groups of healthy mice with or without MAIDS were included as additional controls. The results are shown in Figure 4. As expected, neither RIPK1 nor RIPK3 proteins were detected within MCMV-infected eyes of healthy mice without MAIDS that did not develop retinal necrosis. Similarly, neither RIPK1 nor RIPK3 proteins were detected within mock-infected eyes of mice with or without MAIDS that showed relatively normal retinal architectures. In sharp contrast, both RIPK1 and RIPK3 proteins were detected within MCMV-infected of wildtype MAIDS-10 mice, as well as MCMV-infected eyes of MAIDS-10 mice deficient in either NLRP3, NLRP1b, or AIM2, all of which exhibited an atypical pattern of retinal disease (Figure 4). It is noteworthy that MCMV-infected eyes of immunologically normal mice without MAIDS but nonetheless deficient in either NLRP1b or AIM2 also showed significant production of both RIPK1 and RIPK3 proteins.

## 4. Discussion

Invasion of the retina of an HIV-immunosuppressed patient by HCMV via the vasculature leads to a dramatic necrosis of all layers of the retinal architecture, thereby resulting in a full-thickness retinal necrosis. To better identify those virologic, immunologic, and/or pathogenic events that contribute to the onset and development of full-thickness retinal necrosis during AIDS-related HCMV retinal necrosis, we have employed mice with a retrovirus-induced immunodeficiency (MAIDS) as a mouse model that mimics many of the immunopathogenic features of this sight-threatening retinal disease in AIDS patients [28,29,30]. Because PCD is a component of innate immunity that contributes to host control at the earliest stages of virus infection [11], we have become interested in the possible contributions of these signaling pathways, either individually or through crosstalk, toward the pathogenesis of AIDS-related HCMV retinal necrosis using mice with MAIDS-related MCMV retinal necrosis.

We first explored extrinsic apoptosis (Table 1). This form of apoptosis is often initiated by TNF death receptors (TNFR1), which leads to cell death via caspase-8 autoactivation and subsequent direct activation of caspase-3 [42,43]. Apoptosis is a non-lytic process; apoptotic cells are disassembled into membrane-enveloped fragments that are rapidly cleared by professional phagocytes such as macrophages and dendritic cells. Evidence for the operation of caspase-8-mediated apoptosis during the onset and development of retinal necrosis within whole MCMV-infected eyes of MAIDS-10 mice was suggested by a significant increase in intraocular amounts of TNF, proapoptotic TNF receptors, caspase-8, and caspase-3 by 10 days postinfection, when retinal necrosis development is most severe [10]. Additional studies using MAIDS-10 mice deficient in proapoptotic TNF receptors and unable to execute TNF-mediated extrinsic apoptosis also showed an expected significant decrease in caspase-8 within whole MCMV-infected eyes. This was associated with a concomitant and significant decrease in the frequency of full-thickness retinal necrosis [10]. However, direct examination of retinal tissues from MCMV-infected eyes of wildtype MAIDS-10 mice showing classic full-thickness retinal necrosis and subjected to TUNEL assay for detection and quantification of DNA fragmentation associated with apoptotic cells revealed the number of TUNEL-positive cells distributed throughout the retinal tissue to be remarkably low. This was true even during early stages of retinal necrosis development [10]. Although the use of TUNEL assay as a marker for apoptosis has become controversial due to a better understanding of caspase-8-mediated apoptosis and its now recognized association with necroptosis development [40,41,44], we presently conclude that extrinsic apoptosis contributes minimally to the onset and development of MCMV retinal necrosis during MAIDS-10. One possible explanation for this outcome is the finding that cytomegaloviruses encode for cell death suppressors including those for apoptosis [11]. The MCMV genome encodes for M36, which is an immediate early protein that serves to suppress extrinsic apoptosis by binding directly to procaspase-8 to prevent autoactivation [11]. In this way, it might contribute to the resistance of MCMV-infected retinal cells to caspase-8-mediated apoptotic cell death. It is also noteworthy that HCMV encodes for a homologous protein, UL36, that serves as a caspase-8 inhibitor to suppress extrinsic apoptosis [45] (Table 1).

In sharp contrast to apoptosis, canonical pyroptosis is a lytic PCD that directly stimulates prominent inflammation during virus infection (Table 1). A role for pyroptosis has been implicated in several human disease states, including cancer [46], as well as infectious diseases such as COVID-19 [47]. Moreover, there is increasing evidence that pyroptosis operates during the pathogenesis of several noninfectious retinal diseases, such as age-related macular degeneration, [48], retinitis pigmentosis [49], glaucoma [50], and retinal ischemia [51]. In comparison, there has been a substantial lack of information on a possible role for pyroptosis during the pathogenesis of virus-associated herpesvirus retinal diseases, including HCMV retinal necrosis.

To help fill this knowledge gap, we performed a series of studies reported herein that focused exclusively on inflammasomes. These protein complexes are crucial to the initiation of canonical pyroptosis and pivotal to the induction of inflammation through activation of caspase-1 following assembly of a functional inflammasome complex [16,17]. Because several sensor components of inflammasomes have been recognized depending on which inflammasome sensor is activated, in our study, we included NLRP3 and NLRP1b, which respond to several diverse extracellular stimuli [15,19,20]. We also included AIM2 due to its documented recruitment during invasion of double-stranded DNA viruses [21], including cytomegaloviruses [22]. Our findings have provided new evidence that (i) multiple inflammasomes associated with the initiation of pyroptosis were stimulated within MCMV-infected eyes of wildtype MAIDS-10 mice, as suggested by significantly increased transcription of genes that encode for NLRP3, NLRP1b, and AIM2 inflammasomes; (ii) MCMV-infected eyes of knockout MAIDS-10 mice deficient in either the NLRP3, NLRP1b, or AIM2 inflammasomes did not exhibit the full-thickness retinal necrosis typically observed in MCMV-infected eyes of wildtype MAIDS-10 mice; and (iii) MCMV-infected eyes of MAIDS-10 mice deficient in either the NLRP3, NLRP1b, or AIM2 inflammasomes instead developed an atypical retinal pathology striking for its preservation of the neurosensory retina, which is consistently lost in MCMV-infected eyes of wildtype MAIDS-10 mice and replaced by necrotic tissue during full-thickness retinal necrosis development.

It is noteworthy that these findings are in good agreement with findings from our previous study, which investigated a possible role for caspase-1-mediated pyroptosis during MAIDS-related MCMV retinal necrosis pathogenesis by focusing on several key components of the pathway that function downstream of inflammasome complex formation [31]. Importantly, MCMV-infected eyes of MAIDS-10 mice deficient in either caspase-1, GSDMD, or IL-18 all consistently showed a pattern of retinal disease similar, if not identical, to that observed in the present study for MAIDS-10 mice deficient in the NLRP3, NLRP1b, or AIM2 inflammasomes. Taken together, these results support the hypothesis that caspase-1-mediated pyroptosis, unlike apoptosis, plays a consequential role during the pathogenesis of MAIDS-related MCMV retinal necrosis, especially during destruction of the normal architecture of the neurosensory retina.

Recent work by others has identified an MCMV-encoded protein, M84, that suppresses AIM2 function and thereby counteracts inflammasome activation and caspase-1-mediated pyroptosis. This was suggested by poor replication of M84-deficient MCMV in macrophages due to increased production of proinflammatory cytokines and pyroptosis [52,53]. Whether use of an M84-deficient MCMV in the MAIDS model of MCMV retinal necrosis would affect the typical development of full-thickness retinal necrosis is yet to be determined. In addition, a possible interaction of M84 with NLRP3 and/or NLRP1b inflammasomes as occurs with AIM2 inflammasome remains unclear. It is interesting to note, however, that M84 of MCMV has amino acid homology to UL83 and UL84 proteins of HCMV with HCMV-encoded UL83 also shown to impact AIM2 sensing and signaling [54] (Table 1).

One unexpected outcome of the present investigation was the observation that multiple inflammasomes were stimulated simultaneously within MCMV-infected eyes during the progression of MAIDS-related retinal necrosis. In hindsight, this should not be surprising because we evaluated the entire globe of the virus-infected eye for evidence of stimulation of individual inflammasomes. In mice, as well as in humans, inflammasomes are expressed in various cell types [55], so different cell types can assemble distinct inflammasome complexes with their activation leading to diverse pathogenic outcomes and consequences. Thus, different cell types that collectively compose retinal tissue may use different or even multiple inflammasome complexes in response to virus infection. This possibility has been recently underscored by us through a series of in vitro studies showing that MCMV and HCMV differ in pyroptosis induction in different types of cultured cell lines during productive replication [56]. Our findings presented herein may therefore represent a composite of results that originate from different cell types from which the retinal architecture within the eye is composed. We are presently evaluating different retinal cell types for their inflammasome-associated pyroptosis signatures during the progression of MAIDS-related MCMV retinal necrosis using an immunostaining approach. Special attention is being given to photoreceptors and Muller cells of the neurosensory retina, which are retinal cell types not included in our recent in vitro pyroptosis induction investigation [56].

Unlike apoptosis and pyroptosis, necroptosis is a caspase-independent PCD pathway stimulated by RIPK1 recruitment of RIPK3, which is executed by RIP homotypic interacting motif (RHIM) interactions [39,40,41] (Table 1). Like pyroptosis, necroptosis is a proinflammatory PCD that leads to cell death due to loss of plasma membrane integrity executed by cleavage of the pore-forming protein MLKL, which results in cell swelling, rupture, and the release of cellular contents, which ultimately stimulates inflammation within virus-infected tissue [11,40]. MCMV-encoded M45 protein, however, can prevent necroptosis in mouse cells by disrupting the stimulation of RIPK3 by RIPK1 via interaction with RHIM [45] (Table 1). Necroptosis can proceed as an alternative to extrinsic apoptosis when caspase-8 activity is compromised during infection with cytomegaloviruses that utilize homologous virus-encoded cell death suppressors [43]. Moreover, virus-infected cells may exhibit markers for multiple PCD pathways. This possibility has been referred to as PANoptosis, which is controlled by a hypothetical unifying death complex, the PANosome [57,58]. Although the concept of PANoptosis is controversial, we discovered that both RIPK1 and RIPK3 proteins are stimulated intraocularly in groups of wildtype MCMV-infected eyes of MAIDS-10 mice undergoing caspase-1-mediated pyroptosis as well as in groups of MCMV-infected eyes of MAIDS-10 mice deficient in either NLRP3, NLRP1b, or AIM2 inflammasomes. Taken together, these results suggest that necroptosis and pyroptosis may operate simultaneously during MAIDS-related MCMV retinal necrosis pathogenesis.

Finally, it has not escaped our attention that neutrophils may serve as a source for proinflammatory cell death during the pathogenesis of MAIDS-related MCMV retinal necrosis through the cellular process of NETosis. This process is a noncanonical caspase-11-mediated signaling pathway involving GSDMD [59,60]. Upon neutrophil phagocytosis of Gram-negative bacteria, GSDMD-induced noncanonical pyroptosis has been found to induce neutrophils to release antimicrobial neutrophil extracellular traps (NETs) by plasma membrane rupture [61]. More recently, a role for neutrophil-associated NETosis has been extended to include ocular HSV1 infection and pathogenesis [62]. Because (i) we have evidence that capsase-11-dependent noncanonical pyroptosis operates within MCMV-infected eyes of MAIDS-10 during development of retinal necrosis [31], and (ii) neutrophils are prominent within retinal tissues during development of AIDS-related HCMV retinal necrosis [4,5] as well as MAIDS-related MCMV retinal necrosis [63], we hypothesize that neutrophil-mediated NETosis may also contribute to the evolution of cytomegalovirus retinal disease development. Studies are presently oriented toward testing this exciting hypothesis.

## Figures and Tables

**Table 1:** Comparison of apoptosis, pyroptosis, and necroptosis cell death signaling pathways relative to MCMV and HCMV infection ^a^.

	Signaling Pathway	Consequences	Proinflammatory?	Pathway Suppression via Virus-Encoded Gene ^b^
Extrinsic Apoptosis	TNFR1	Cell lysis		M36 (MCMV)
	TNF	Phagocytosis	No	UL36 (HCMV)
	Caspase-8			
	Caspase-3			
Canonical Pyroptosis	Inflammasomes	Cell lysis		M84 (MCMV)
	Caspase-1	Release of	Yes	UL83 (HCMV)
	Gasdermin D			
Necroptosis	RIPK1	Cell lysis		M45 (MCMV)
	RIPK3	Release of	Yes	
	MLKL	cellular contents		

^a^ Modified from Chien et al. [10]; ^b^ summarized by Mocarski [11].

**Table 2:** Frequency of full-thickness retinal necrosis within MCMV-infected eyes of MAIDS-10 mice at 10 days postinfection.

Wildtype MAIDS-10	89% (8/9)
NLRP3^−/−^ MAIDS-10	0% (0/4)
NLRP1b^−/−^ MAIDS-10	0% (0/4)
AIM2^−/−^ MAIDS-10	0% (0/4)

## Data Availability

The original contributions presented in the study are included in the article. Further inquiries can be directed to the corresponding author.
